# Draft Genome Sequences of Two *Bacillus thuringiensis* Strains and Characterization of a Putative 41.9-kDa Insecticidal Toxin

**DOI:** 10.3390/toxins6051490

**Published:** 2014-04-30

**Authors:** Leopoldo Palma, Delia Muñoz, Colin Berry, Jesús Murillo, Primitivo Caballero

**Affiliations:** 1 Instituto de Agrobiotecnología, Consejo Superior de Investigaciones Científicas-Universidad Pública de Navarra-Gobierno de Navarra, Campus Arrosadía, Mutilva Baja, Navarra 31192, Spain; E-Mail: leopoldo.palma@unavarra.es; 2 Grupo de Protección Cultivos, Departamento de Producción Agraria, Escuela Técnica Superior de Ingenieros Agrónomos, Universidad Pública de Navarra, Pamplona, Navarra 31006, Spain; E-Mails: dmunoz@unavarra.es (D.M.); jesus.murillo@unavarra.es (J.M.); 3 Cardiff School of Biosciences, Cardiff University, Park Place, Cardiff CF10 3AT, UK; E-Mail: Berry@cf.ac.uk

**Keywords:** *Bacillus thuringiensis*, insecticidal toxins, next-generation sequencing, genome annotation, microbial control, insecticidal activity

## Abstract

In this work, we report the genome sequencing of two *Bacillus thuringiensis* strains using Illumina next-generation sequencing technology (NGS). Strain Hu4-2, toxic to many lepidopteran pest species and to some mosquitoes, encoded genes for two insecticidal crystal (Cry) proteins, *cry1Ia* and *cry9Ea*, and a vegetative insecticidal protein (Vip) gene, *vip3Ca2*. Strain Leapi01 contained genes coding for seven Cry proteins (*cry1Aa*, *cry1Ca*, *cry1Da*, *cry2Ab*, *cry9Ea* and two *cry1Ia* gene variants) and a *vip3* gene (*vip3Aa10*). A putative novel insecticidal protein gene 1143 bp long was found in both strains, whose sequences exhibited 100% nucleotide identity. The predicted protein showed 57 and 100% pairwise identity to protein sequence 72 from a patented Bt strain (US8318900) and to a putative 41.9-kDa insecticidal toxin from *Bacillus cereus*, respectively. The 41.9-kDa protein, containing a C-terminal 6× HisTag fusion, was expressed in *Escherichia coli* and tested for the first time against four lepidopteran species (*Mamestra brassicae*, *Ostrinia nubilalis*, *Spodoptera frugiperda* and *S. littoralis*) and the green-peach aphid *Myzus persicae* at doses as high as 4.8 µg/cm^2^ and 1.5 mg/mL, respectively. At these protein concentrations, the recombinant 41.9-kDa protein caused no mortality or symptoms of impaired growth against any of the insects tested, suggesting that these species are outside the protein’s target range or that the protein may not, in fact, be toxic. While the use of the polymerase chain reaction has allowed a significant increase in the number of Bt insecticidal genes characterized to date, novel NGS technologies promise a much faster, cheaper and efficient screening of Bt pesticidal proteins.

## 1. Introduction

*Bacillus thuringiensis* (Bt) is one of the best-characterized entomopathogenic bacteria, with many strains bearing a wide variety of insecticidal genes [1]. Delta-endotoxins (Cry and Cyt proteins), synthesized during the stationary growth phase as crystalline, parasporal inclusions, are highly active against a wide range of insects [1,2] but also against nematodes [3] and a human-pathogenic protozoan [4]. Parasporins (Ps), another crystal protein group of Bt, may possess the typical three-domains of Cry proteins (Ps1 and Ps3 but not Ps2 or Ps4) and, although lacking insecticidal activity, exhibit strong cytocidal activity against human cancer cells of various origins upon protease activation [5,6]. Crystal proteins accumulate in the cells as parasporal inclusions and can account for up to 25% of the sporulated cell dry weight. Under laboratory conditions, Bt is able to produce nearly 0.5 mg of crystal proteins per mL [7]. In order to produce this large amount of protein and form crystals, some insecticidal proteins require the presence of helper proteins, *i.e.*, P19 and P20 for the stable production of Cyt1A and Cry11A [8]. P20 also enhances the expression and crystallization of Cry1Ac in acrystalliferous and plasmid-negative Bt strains [9] and synergizes the toxicity of Cry11A against third-instar larvae of *Aedes aegypti* (Diptera: Culicidae) [10]. Bt synthesizes additional insecticidal proteins during the vegetative growth phase, which are subsequently secreted into the growth medium. These proteins are commonly known as vegetative insecticidal proteins (Vips) and hold insecticidal activity against lepidopteran [11], coleopteran [12] and some homopteran pests [13]. A less characterized secretory protein with no amino acid similarity to previously known Vips, and termed Sip protein (secreted insecticidal protein), has insecticidal activity against coleopteran pests [14]. The only Sip protein discovered to date is a 41-kDa protein, with high toxicity against *Leptinotarsa decemlineata* (Coleoptera: Chrysomelidae) and inhibits larval development of *Diabrotica undecimpunctata howardi* and *Diabrotica virgifera virgifera* (Coleoptera: Chrysomelidae) [14]. Bt also produces Mtx-like and Bin-like proteins, which share amino acid similarities with mosquitocidal Mtx and Bin toxins, respectively, from *Lysinibacillus sphaericus* (formerly *Bacillus sphaericus*) [15,16]. In summary, Bt insecticidal proteins may be classified into at least five distinct protein groups according to their amino acid identity and protein structure: Bin-like, three-domain Cry toxins, Cyt, Mtx-like, Sip, and Vip proteins [16]. 

In addition to the insecticidal toxins mentioned above, some *B. thuringiensis* strains produce other virulence factors such as phospholipases, proteases, haemolysins, enterotoxins, enhancin-like proteins (metalloproteases) and chitinases, plus extracellular (secreted) antimicrobial compounds (AMPs) such as bacteriocins and β-exotoxins, which may contribute to the pathogenicity of this bacterium against insects [1]. The phospholipases, haemolysins, enhancin-like proteins and chitinases are synthesized in the insect midgut during vegetative growth, facilitating the dissemination of the bacteria through the peritrophic membrane and towards the hemocoel [1,17]. The β-exotoxins, also known as thuringiensins, are secreted, heat-stable, secondary metabolites analogues to the nucleotide adenine and with low molecular weight (~700 Da) [18].

Screening programs have identified thousands of Bt strains and insecticidal genes active against a wide range of insect orders and several nematodes, mites and protozoans [16]. However, such screening programs have been usually costly and lengthy and not always efficient. Generally, toxin genes are identified by polymerase chain reaction (PCR) analyses, along with restriction fragment length polymorphism (RFLP) analysis of the amplicons, followed by time-consuming chromosome-walking techniques to recover the full-length coding sequence of each toxin [19,20,21]. Today, next-generation sequencing technologies (NGS) allow rapid sequencing of entire genomes with profitable cost-effective ratios [22,23]. This study aimed to sequence and analyze the genome of two Bt strains containing known insecticidal genes using Illumina NGS-sequencing technology and to characterize a putative 41.9-kDa insecticidal protein of unreported biological activity.

## 2. Materials and Methods

### 2.1. Bacterial Strains and Plasmids

The Bt strains used in this work belong to a collection maintained at the Universidad Pública de Navarra (Pamplona, Spain) which comprises more than 2000 strains organized in two major sub-collections: one from mainland Spain and a second one from the Canary Islands [24,25]. Strains Hu4-2 and Leapi01 are from the Spanish mainland sub-collection. Strain Leapi01 was isolated from a dead *Mythimna loreyi* (Lepidotera: Noctuidae) larva during a natural epizootic in corn crops in the province of Badajoz (Spain) [26]. Strain Hu4-2 was isolated from dust samples obtained in a maize grain silo from the province of Huesca (Spain) [27]. Bt strains and *E. coli* DH5α, used for routine DNA manipulations, were cultured in Luria-Bertani (LB) medium (1% tryptone, 0.5% yeast extract, and 1% NaCl, pH 7.0) at 28 °C and 37 °C, respectively. Strain *E. coli* BL21(DE3) was used for protein expression and was propagated in 2× YT medium (1.6% tryptone, 1% yeast extract, and 0.5% NaCl, pH 7.0) at 37 °C. Vectors pGEM-T Easy (Promega) and pET-28b(+) (Novagen) were used for routine cloning of PCR products and for the production of recombinant protein, respectively.

### 2.2. DNA Isolation, Sequencing and Computational Analysis

For genome sequencing, total DNA (chromosome and plasmids) was isolated using the Wizard Genomic DNA Purification Kit (Promega) following the instructions for DNA isolation from Gram-positive bacteria. Purified total DNA from strain Hu4-2 was used to construct a pooled Illumina library and sequenced on a HiSeq 2000 Sequencing System (Illumina Sequencing) in a single read mode with a read length of 50 bases (GATC Biotech, Konstanz, Germany). Reads were then assembled using the CLC Genomic Workbench software (CLC Bio, Aarhus, Denmark) with the *de novo* assembly tool and default parameters. Purified total DNA from Leapi01 was sequenced at the Beijing Genomics Institute (BGI, Shenzhen, China) using high-throughput Illumina sequencing technology and a paired-end library sequencing of 500 bp. Reads were assembled using SOAP denovo (version 1.05) with an optimized k-mer size of 59-mer. Identification and annotation of insecticidal genes was done with BLAST [28] using custom insecticidal toxin databases and the BtToxin_scanner [29], and complemented with the NCBI Prokaryotic Genome Annotation Pipeline (released 2013) and the RAST server [30]. The custom insecticidal toxin database was constructed with amino acid sequences of Cry and Cyt proteins [1], mosquitocidal proteins Mtx1 and Mtx2 [15], the secreted insecticidal protein Sip [14], vegetative insecticidal proteins Vip1/Vip2 and Vip3 [11,12], cancer cell-killing Cry proteins or parasporins [5,6], accessory proteins P19 and P20 [8] and the haemolytic enterotoxin HBL from *Bacillus cereus* [31]. Multiple sequence alignments, signal peptide and conserved domain analyses plus primer design were performed using suitable tools included in Geneious Pro v6.1.4 [32]. 

### 2.3. Amplification and Cloning of the Full-Length 41.9-kDa-Protein Gene

Primers were designed to amplify the full-length CDS sequence of the putative 41.9-kDa protein but excluding the stop codon, allowing the 6× HisTag fusion of the pET-28b(+) vector into the 3’ terminus of the gene. Forward and reverse primers included a 5’-*Nco*I and a 3’-*Sal*I restriction sites, respectively. The PCR reaction was performed in a 25 μL reaction mixture containing 5 μL 5× reaction buffer, 10 mM dNTP mixture, 6.3 pmol each of forward and reverse primers, 0.5 U proof reading PrimeSTAR HS DNA polymerase (Takara) and 100 ng total DNA in an C1000 Touch thermal cycler (Bio-Rad) using the following cycling conditions: 4 min initial denaturation at 94 °C, 35 amplification cycles (1 min denaturation at 94 °C, 1 min annealing at 52 °C and 72 s extension at 72 °C) and a final extension step at 72 °C for 10 min. Electrophoresed PCR products (1% agarose gels) were gel-purified using NucleoSpin Extract II kit (Macherey-Nagel), end-modified by an A-tailing procedure and ligated into pGEM-T easy according to the manufacturer’s instructions (Promega). Ligation mixtures were then transformed into *E. coli* DH5α using standard procedures [33]. Plasmid DNA was purified using the NucleoSpin Plasmid kit (Macherey-Nagel) and sequenced (Sistemas Genómicos, Valencia, Spain). A clone containing an insert identical to the genome sequence of the 41.9 kDa protein CDS was selected, the insert was excised by digestion with *Nco*I and *Sal*I, purified from the agarose gel, ligated into predigested pET-28b(+) and transformed into *E. coli* BL21(DE3).

### 2.4. Expression and Purification of the 41.9-kDa Recombinant Protein

An *E. coli* BL21(DE3) clone harboring the pET28-b(+) vector containing the 41.9-kDa protein CDS sequence was pre-cultured overnight at 37 °C and 200 rpm in 2× YT medium containing 50 μg/mL kanamycin. A 1/25 dilution of this pre-culture was inoculated into 1 liter 2× YT medium containing 50 μg/mL kanamycin and further incubated with vigorous shaking (250 rpm) until OD600 = 0.7−1.0. Expression was induced immediately with 1mM isopropyl-β-D-1-thiogalactopyranoside (IPTG, final concentration) and the culture was incubated for a further 16 h at 37 °C. Samples were centrifuged at 5000 g for 15 min at 4 °C and the resulting pellet was weighed and resuspended in 3 mL sonication buffer per gram of pellet, containing 20 mM sodium phosphate buffer pH 7.4, 0.5 M NaCl, 3 mg/mL lysozyme, 25 U/μL Benzonase (Novagen) per mL of buffer and 100 μM phenylmethylsulfonyl fluoride (PMSF). Samples were further incubated at 37 °C for 1 h and sonicated on iced-water with a Branson Analog Sonifier S-250 (Fisher Scientific) by applying two 1-min pulses with 60% constant duty cycle and separated by a 1-min cooling period. Insoluble material was removed by centrifugation at 12,000 g for 30 min at 4 °C and the soluble cellular fraction filtered through sterile 0.45 and 0.22 μm syringe filters. Protein purification was performed at room temperature (RT) using Protino Ni-TED 2000 Packed Columns according to the manufacturer’s instructions (Macherey-Nagel). Once the bound polyhistidine-tagged protein was eluted, the buffer exchange procedure was performed immediately at room temperature with Milli-Q water and GE Healthcare PD-10 desalting columns to prevent protein aggregation and precipitation. This step also prevents the potential toxic effects of imidazole [34,35] to the insects tested in bioassays. The resultant expressed protein was then analyzed by sodium dodecyl sulfate-polyacrylamide gel electrophoresis (SDS-PAGE), stained with Coomassie brilliant blue R-250 (Sigma-Aldrich). Duplicate unstained Coomassie SDS-PAGE gel was also analyzed by Western Blot using anti-His-tag antibodies (Sigma-Aldrich, Seelze, Germany) [33]. The protein concentration was quantified by the Bradford method [36].

### 2.5. Insect Rearing and Bioassays

For novel putative toxins of unknown biological activity, the highest protein concentrations (limited by amount of protein produced in expression experiments) were used in bioassays to detect any sign of toxicity (e.g., mortality or impaired growth) for the species tested. If toxicity was detected, we carried out bioassays using lower protein concentrations in order to establish dose-mortality responses and LC_50_ (mean lethal concentration) values. For qualitative bioassays with *Myzus persicae* (Homoptera: Aphididae), cohorts of second instar nymphs were obtained from gravid females during a two-day period prior to the experiment. To evaluate insecticidal activity, the purified (soluble) recombinant protein was incorporated into a liquid diet containing 20% (w/v) sucrose in Milli-Q water at a single high concentration of 1.5 mg/mL. Fifteen-second instar nymphs were placed inside a cylindrical plastic cage without lid (3 cm diameter × 1.5 cm height). The cages were then sealed with a Parafilm layer, on which 100 µL drops of protein-diluted diet or protein-free diet (as negative controls) were loaded and confined using a second Parafilm layer, then aphids were allowed to feed through the Parafilm membrane. Bioassays were conducted at 25 °C, 60 ± 5% RH, and a 16:8 h light:dark photoperiod and mortality was recorded after 72 h. For other bioassays, 24 neonate larvae of four lepidopteran species, one from the Pyralidae family, *Ostrinia nubilalis*, and three from the Noctuidae family, *Mamestra brassicae*, *Spodoptera frugiperda* and *S. littoralis*, were used. The purified (soluble) recombinant protein diluted in water was poured onto each well of 24-well plates (35 µL/well) containing a layer of artificial diet and allowed to dry at a final concentration of 4.8 µg/cm^2^ and one neonate larvae per well. Water was also used instead of protein dilutions in negative controls and the bioassay was repeated twice. Bioassays were conducted at 25 °C, 60% ± 5% RH, and a 16:8 h light:dark photoperiod and mortality was recorded after seven days. 

### 2.6. Nucleotide Sequences Accession Numbers

Draft genome sequences have been deposited in GenBank under accessions AMXT00000000 (GenBank BioProject PRJNA175233) and AMXS00000000 (Genbank BioProject PRJNA175222) for strains Hu4-2 and Leapi01 strains, respectively.

## 3. Results and Discussion

Finding novel proteins is particularly important to manage the increasing resistance occurrence to Bt-based insecticides or Bt-plants reported for some species [37,38,39]. Today, next-generation sequencing (NGS) technologies [22,23,40,41,42] arise as a novel and useful tool for the discovery of completely novel insecticidal-toxin genes that would otherwise be difficult to identify. NGS technologies provides a fast and reliable framework to obtain complete genomic sequences, and offers excellent cost-effective ratios since complete genomes sequences can be obtained for less than 10^−4^ euro per nucleotide. In this work, an efficient strategy combining Illumina *de novo* sequencing and a genome annotation pipeline, which allowed the rapid identification of all kinds of insecticidal genes in sequenced Bt strains, has been performed, and a novel putative 41.9-kDa insecticidal protein analyzed. 

### 3.1. Genome Assembly and Annotation of Bt Strains Hu4-2 and Leapi01

As many as 393 contigs were obtained from strain Hu4-2 totaling 6,458,628 bp with the N50 value [43] at 43,448 bp and 34.7% G+C content. Genome annotation produced 6888 coding sequences (CDSs) with 44 pseudogenes and 37 RNA genes (ribosomal and tRNAs). The assembly contained two complete insecticidal crystal protein genes (*cry1Ia7* and *cry9Ea*), a vegetative insecticidal protein gene (*vip3Ca2*) (Table 1), which was flanked by putative transposase genes (data not shown), and a CDS coding for a protein with 62% pairwise identity to a putative 41.9-kDa insecticidal toxin from *B. cereus* (Table 1). The draft genome for strain Hu4-2 also included short incomplete sequences exhibiting homology to several Cry toxins (Table 1). We do not know whether these incomplete sequences represent true incomplete genes or are the result of misassemblies, which could be due to the existence of regions of high identity among different insecticidal genes and the short read lengths. In addition, this strain also contained two short vestigial toxin gene remnants (VTGR) or pseudogenes (Table 1), with internal stop codons and overlapping BLASTx hits in different frames (frame-shifts), exhibiting 98% and 46% pairwise identity to a *cry2Ab* and *vip3Ba* genes. Two CDSs with homology to the helper protein P19 (accession No. AAB34196) were also found exhibiting 44% and 47% pairwise identity and located upstream of incomplete (lacking the 3’ end) *cry1Ca* and *cry1Fa* sequences, respectively. The complete haemolysin (*hbl*) coding operon from *B. cereus* was also found. The Hu4-2 assembly showed no homology with other toxins such as Mtx toxins, Vip1/Vip2, Sip or parasporin proteins. 

**Table 1:** Insecticidal proteins showing homology with the draft genomes of Bt strains Hu4-2 and Leapi01.

Strain	Closest homolog	Accesion number	% aa identity	CDS completeness
Hu4-2	Cry1Ad	AAA22340	100	Incomplete
	Vip3Ba	AAV70653	46	VTGR
	Cry9Ea	BAA34908	100	Full-length
	Cry1Ab	AAA22330	100	Incomplete
	Cry1Ad	AAA22340	100	Incomplete
	Cry1Fa	AAA22348	100	Incomplete
	Vip3Ca2	AEE98106	100	Full-length
	41.9 kDa	ZP_04231421	62	Full-length
	Cry1Da	CAA38099	100	Incomplete
	Cry1Ia7	AAM73516	100	Full-length
	Cry1Ca+P19	CAA30396	100	Incomplete
	Cry1Aa	AAA22353	100	Incomplete
	Cry1Ca	CAA30396	100	Incomplete
	Cry1Fa	AAA22348	100	Incomplete
	Cry1Fa	AAA22348	100	Incomplete
	Cry1Ea	CAA37933	99	Incomplete
	Cry1Da	CAA38099	100	Incomplete
	Cry2Ab	ACC86136	98	VTGR
	Cry1Fa+P19	AAA22348	100	Incomplete
	Cry1Ab	AAA22330	100	Incomplete
	Cry1Ad	Q03744	99	Incomplete
	Cry1Fa	AAA22348	100	Incomplete
Leapi01	Cry1Aa	AAA22330	99	Incomplete
	Cry1Ab	AAA22330	100	Incomplete
	Cry9Ea	CAA85764	100	Full-length
	Cry1Ca	CAA30396	100	Full-length *
	Cry1Da	CAA38099	100	Full-length *
	Cry1Ia2	CAA44633	100	Full-length
	Cry1Ab	AAA22330	100	VTGR
	Cry1Aa	AAA22353	100	Incomplete
	Cry1Ab	AAA22330	100	Incomplete
	41.9-kDa	ZP_04231421	62	Full-length
	Vip3Aa10	AAC37036	99	Full-length
	Cry2Ab	AAA22342	100	Full-length
	Cry1Aa	BAA00257	100	Full-length **
	Cry1Ia14	ACG63871	100	Full-length **

Notes: Incomplete: unfinished contig sequences; VTGR: vestigial toxin gene remnants or pseudogenes; * Split by N-in-frame characters, the full-length coding sequence was deduced by alignment; ** The full-length coding sequence was re-constructed by re-assembly with plasmid pCT281 sequence; Acc. num.: GenBank accession number.

The Leapi01 draft genome produced 225 scaffolds totaling 6,156,720 bp with the N50 value at 31,921 bp and 34.8% G+C content. Genome annotation identified 6158 CDSs with 40 pseudogenes and 41 RNA genes. This strain carried seven complete insecticidal crystal protein genes (*cry1Aa*, c*ry1Ca*, *cry1Da*, *cry2Ab*, *cry9Ea* and two *cry1Ia* variants) and a *vip* gene (*vip3Aa10*) that, as occurred with the *vip3Ca2* gene of strain Hu4-2, was flanked by two sequences encoding putative transposases (data not shown). This strain also contained the CDS coding for the putative 41.9-kDa insecticidal toxin found in Hu4-2 strain (Table 1) and the complete haemolysin (*hbl*) coding operon previously found in strain Hu4-2. Genes *cry1Ca*, *cry1Da* and *cry1Ia2* were located closely together into the same scaffold, which also encoded a putative helper protein with 47.7% identity to P19 (accession No. AJ010753). Scaffold 118 (18,932 bp) exhibited 99% pairwise identity with plasmid pCT281 (281, 231 bp) from Bt strain CT-43. To evaluate the possibility that strain Leapi01 could contain a plasmid similar to pCT281, we performed a reassembly of the Leapi01 reads using pCT281 as reference sequence. This resulted in a consensus sequence of 281,256 bp showing synteny and 98.7% pairwise identity to pCT281. This consensus included genes *cry1Aa3*, *cry1Ia14*, *cry2Ab* and *vip3Aa10*; however, gene *cry2Aa9* was not included in this or any of the other contigs of the assembly, although it is present in plasmid pCT281. In addition to the toxin genes described above, this strain also contained short contigs with homology to diverse *cry1A* genes and a short VTGR sequence with homology to *cry1Ab* gene (Table 1). This strain did not contain sequences with homology to Mtx toxins, Vip1/Vip2, Sip or parasporins. Finally, the *vip3* genes from strains Hu4-2 and Leapi01 are preceded by a CDS coding for a trans-acting positive regulator protein (Multiple Virulence Gene Regulator, Mga from group A *Streptococcus*) with 94.9% amino acid identity between both strains [44].

### 3.2. Molecular Characterization of the Putative 41.9-kDa Insecticidal Protein

**Figure 1:**
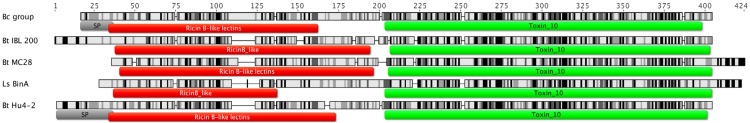
Comparison between *B. cereus* group (Bc) hypothetical protein homologous to 41.9-kDa proteins, *B. thuringiensis* (Bt) 41.9-kDa insecticidal protein from strains IBL 200, MC28 and Hu4-2 (identical to that of strain Leapi01), and *L. sphaericus* (Ls) BinA protein. (SP) predicted signal peptide. Sequence identity is indicated by shading: black for 100%, dark grey for 80%–100%, light grey for 60%–80%, and white for less than 60% identity. Scale indicates residue numbers in the multiple sequence alignment.

The 41.9-kDa genes encoded by Leapi01 and Hu4-2 strains were identical and 1143 bp long, with a deduced amino acid sequence of 380 residues and predicted molecular weight of 41.9 kDa. Their predicted protein sequences exhibited 57 and 100% pairwise identity to protein sequence 72 from patent US8318900 and the 41.9-kDa component of a putative insecticidal toxin from *B. cereus*, respectively. They were identical to the hypothetical protein IE5_04900 from *B. cereus* BAG3X2-2 and showed 38 and 60% identity to BinA and BinB, respectively, the two components of the Bin binary toxin from *L. sphaericus* (Bacillales: Planococcaceae). Protein analysis with Geneious Pro detected a putative signal peptide sequence from residues 1 to 35 (residue positions of the 41.9-kDa protein sequence) plus two conserved domains: a ricin B-like lectin (InterPro family IPR000772) and a Toxin_10 conserved domain commonly found in some binary insecticidal crystal toxins (InterPro family IPR008772) from residues 33 to 153 and from 183 to 377 (residue positions of the 41.9-kDa protein sequence), respectively (Figure 1).

### 3.3. Insecticidal Bioassay of the Putative 41-kDa Insecticidal Protein

**Figure 2:**
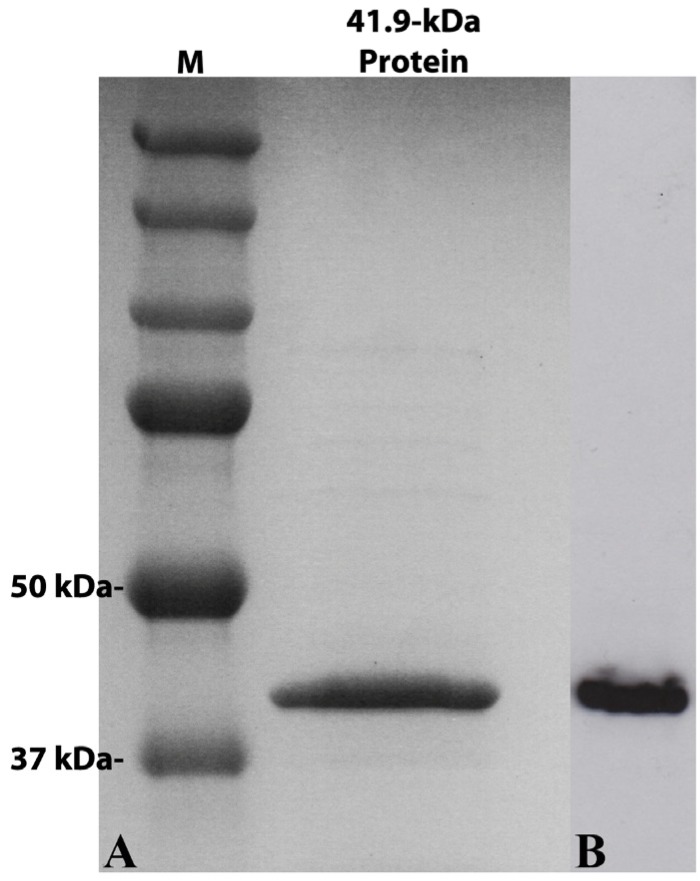
SDS-PAGE gel (**A**) and Western Blot (**B**) analysis of the 41.9-kDa recombinant protein expressed in *E. coli* (right lane). The recombinant protein was tagged with a 6× His in its C-terminal end and purified from a nickel column before separation by electrophoresis; after transfer to a nylon membrane, the protein was detected using anti-His-tag antibodies. A molecular weight marker (Precision plus protein dual color standards, Bio-Rad) was electrophoresed along with the sample and the sizes of the fragments are indicated to the left of the panel.

The gene for the 41.9-kDa protein from strain Hu4-2 was successfully expressed in *E. coli* and produced a major protein of the expected size (approx. 42 kDa) in a soluble form (Figure 2), as observed in SDS-PAGE analysis. At the concentrations used, the purified (soluble) recombinant 41.9-kDa protein caused no mortality or impaired growth of any of the lepidopteran insects tested, namely: *M. brassicae*, *O. nubilalis*, *S. frugiperda* and *S. littoralis*, nor of the green-peach aphid *M. persicae*.

## 4. Conclusions

In this work, we report the draft genome sequences of the Bt strains Hu4-2 and Leapi01, which have been previously characterized with different levels of description [27,45]. Their insecticidal gene content was explored mainly using the BtToxin_scanner [29] and custom BLAST searches [28]. The BtToxin_scanner has previously been demonstrated to be an efficient tool for the identification of insecticidal toxin coding sequences over both nucleotide and protein sequences [29]. It also detected both short vestigial and full-length toxin-coding sequences even with in-frame ambiguous (e.g., N) characters. This situation occurs when NGS data are assembled with sequencing data obtained from pair-end libraries (*i.e.*, Leapi01 strain), which allows for the assessment of the order, distance and orientation of contigs along with their re-assembly into larger sequences or scaffolds [46]. These contigs might contain gaps, which are filled with as many Ns as the most likely estimate of their length [47]. However, the inability of the scanner to detect other valuable proteins (*i.e.*, Sip, Bin, Mtx-like toxins and accessory P19/P20 proteins) made the combination of both methods (custom BLAST searches and scanning) the best approach. Annotation data indicated that even though both strains showed different toxin repertoires, they shared three complete genes: the insecticidal crystal protein gene *cry9Ea*, the putative 41.9-kDa insecticidal protein gene and different *cry1Ia* gene variants (Table 1). The two strains also showed several short sequences encoding toxin fragments (Table 1) of two types: those likely located in partially sequenced or unfinished contigs, commonly found in unclosed draft genomic sequences and short sequences of vestigial toxin gene remnants or pseudogenes, as seen in previous sequencing studies [48]. From the available data, it is not possible to conclude whether the partial sequences represent fragments of whole CDSs or pseudogenes. The pseudogenes, representing residual coding sequences, which seem to be the leftovers of genes undergoing recombination or transposition events, especially when flanked by mobile genetic elements, may constitute proof of toxin evolution and explain the distribution of genes in different strains [49]. In fact, full-length *vip3* genes (*vip3Aa10* and *vip3Ca2*) were also found flanked by short sequences (100 to 300 bp) with homology to putative transposases. In addition, upstream of these genes can be found a conserved CDS with homology to a trans-acting positive regulator protein (Mga), a DNA-binding protein that activates the expression of several important virulence genes in group-A *Streptococcus* [50]. The high similarity of contig 188 from the Leapi01 strain with plasmid pCT281 (~99%), from the Bt strain CT-43 [51], indicated the likely presence of this plasmid in Leapi01. In addition, genes *cry1Aa3*, *cry1Ia14* and *cry2Aa9* have been found close together in a 28,171 bp region of pCT281, constituting a pathogenicity island in strain CT-43 [51]. The other nine-plasmid sequences described for this strain were also screened in Leapi01 or Hu4-2, but they were not detected. Most of the insecticidal genes present in both strains (except that coding for the 41.9-kDa protein) were located in contigs or scaffolds with the highest levels of read coverage and without mapping over reference Bt genomes, indicating they are probably present in high-copy plasmids. However, our work was focused primarily in finding full-length insecticidal genes for cloning independently of their location (plasmid or chromosome). Bt has been considered a safe and environmentally friendly bacterium for the control and management of a wide number of the most damaging insect pests worldwide because of its lack of toxicity and pathogenicity against mammals [52]. However, some strains (*i.e.*, Bt strain 97-27) carry chromosome-encoded virulence factors commonly found in the mammalian opportunistic pathogen, *B. cereus*, that threaten its safety record [53]. Bt is closely related to *B. cereus* and *B. anthracis* [54], and strain 97-27 bears the *hbl* operon, with haemolytic enterotoxin genes *hblCDBA*, which is suspected to cause diarrhea by *B. cereus* contaminated food. Nevertheless, some strains PCR-positive for the *hblA* gene (*i.e.*, Bt strain WS2623) are not cytotoxic or haemolytic, suggesting that this gene is present but incomplete or silent, whereas others, despite their demonstrated safe use as biopesticide, produced the enterotoxin HBL [31]. In this work, both strains exhibited the complete homologous *hbl* operon, but they have not been tested yet for haemolytic activity. The predicted 41.9-kDa protein resembles previously known insecticidal toxins and shares 38 and 60% maximum pairwise identity to components A and B of Bin binary toxin from *L. sphaericus*, respectively. This *L. sphaericus* binary toxin is highly toxic against mosquitoes [15]. Both components are co-transcribed from a single operon before the end of the exponential growth and sporulation as a crystal toxin [15]. The Bin protein sequences are extremely conserved between strains and only five variants have been reported to date with a few differences in their amino acid sequences [55]. In contrast, the 41.9-kDa-protein gene from the Spanish strains was found as a single coding sequence and its deduced amino acid sequence was poorly conserved among those of different Bt strains (34% maximum pairwise identity), in which 41.9-kDa related proteins were also found as single coding sequences. These findings strongly suggested that this protein might have a non-binary behavior and work as a single toxin. When compared with the *L. sphaericus* BinB protein, the 41.9 kDa protein shared the two conserved domains but not the predicted signal peptide and the transmembrane domain, which were found exclusively in the 41.9-kDa protein from the Spanish strains (Figure 1) and suggest that it might be secreted. In fact, this protein has not been detected in previous SDS-PAGE analyses of Leapi01 and Hu4-2 crystal proteins, consistent with synthesis during the vegetative growth phase and subsequent secretion into the growth medium [26,27]. A similar situation occurs with other secreted insecticidal proteins such as Vip3 [11] and Cry1Ia7 proteins [56]. To our knowledge, this study represents the first bioassay of the 41.9-kDa putative insecticidal proteins against any insect targets. Despite the identities with known toxins and the possibility that this 41.9-kDa protein may behave as a non-binary Bt toxin, it caused no mortality or symptoms of impaired growth at very high concentrations against any of the insects tested. This may be because they are outside of the insect’s target range of the protein; the protein may require an unknown second component; the proteolytic activation after secretion (removal of the signal peptide) may occur in Bt but not in *E. coli*; or the protein may not, in fact, be toxic. Other species, including some from different insect orders (*i.e.*, Coleoptera and Diptera), will need to be tested to evaluate the insecticidal potential of this protein. Purified crystals from strain Hu4-2 were previously reported to be highly active against several lepidopteran species (*H. armigera*, *S. exigua*, *S. frugiperda* and *S. littoralis*), moderately toxic for two species of mosquitoes, *Culex pipiens pipiens* (Diptera: Culicidae) and *A. aegypti*, and not active against *Anopheles stephensi* (Diptera: Culicidae) [27]. Likewise, *H. armigera* and *S. littoralis* were very susceptible to the purified crystals of Leapi01 [26]. However, their complete toxin gene contents (Table 1) imply that both strains may have wider host ranges. A previous PCR screening of insecticidal toxins in strain Hu4-2 [27] detected eight *cry* gene fragments (*cry1A*b, *cry1Ad*, *cry1C*, *cry1D*, *cry1F*, *cry1I*, *cry9E*, and *cry2Ab*), although SDS-PAGE analyses suggested that some of them are not expressed. Among the diverse crystal toxin genes found in the Hu4-2 draft genome sequence obtained in this study, we found a complete CDS for only genes *cry9Ea* and *cry1Ia7*, whereas a *cry2Ab* homolog corresponded to a pseudogene with internal stop codons and multiple frame-shifted open reading frames. Sequencing may explain also why 130 to 145-kDa-crystals toxins were detected; these may be likely the product of *cry9* expression (~129.9 kDa). Similarly, the PCR screening of Leapi01 [26] detected six *cry* gene fragments (*cry1Aa*, *cry1Ab*, *cry1Ca*, *cry1Da*, *cry2Ab* and *cry1Ia*) while analysis of the Leapi01 draft genome sequence, revealed seven full-length gene sequences; *cry1Aa*, *cry1Ca*, *cry1Da*, *cry2Ab*, *cry9E* plus two *cry1Ia* variants (*cry1Ia7* and *cry1Ia14*). These results indicated that these PCR analyses missed *cry9Ea* and misrepresented the presence of a unique *cry1Ia* gene variant; additionally, although *cry1Ab* was detected by PCR, annotation of the genome indicated that it was encoded by vestigial (incomplete) gene coding sequence. These previous studies [26,27] also failed to detect the 41.9-kDa proteins genes in both strains, obviously due to the lack of specific PCR primers for molecular analysis. Therefore, with the advent of NGS technology, the screening of novel or previously described holotype toxin genes has become much cheaper, faster and efficient, and may provide additional valuable information on the structural organization and the presence of other proteins such as toxin-assisting chaperones and relevant non-coding sequences (promoters, ribosomal-binding sites, *etc*). 
